# Role of transporters in regulating mammalian intracellular inorganic phosphate

**DOI:** 10.3389/fphar.2023.1163442

**Published:** 2023-03-30

**Authors:** Michael L. Jennings

**Affiliations:** Department of Physiology and Cell Biology, University of Arkansas for Medical Sciences, Little Rock, AR, United States

**Keywords:** inorganic phosphate, transport, regulation, SLC20, XPR1, inositol pyrophosphates

## Abstract

This review summarizes the current understanding of the role of plasma membrane transporters in regulating intracellular inorganic phosphate ([Pi]_In_) in mammals. Pi influx is mediated by SLC34 and SLC20 Na^+^-Pi cotransporters. In non-epithelial cells other than erythrocytes, Pi influx *via* SLC20 transporters PiT1 and/or PiT2 is balanced by efflux through XPR1 (xenotropic and polytropic retrovirus receptor 1). Two new pathways for mammalian Pi transport regulation have been described recently: 1) in the presence of adequate Pi, cells continuously internalize and degrade PiT1. Pi starvation causes recycling of PiT1 from early endosomes to the plasma membrane and thereby increases the capacity for Pi influx; and 2) binding of inositol pyrophosphate InsP8 to the SPX domain of XPR1 increases Pi efflux. InsP8 is degraded by a phosphatase that is strongly inhibited by Pi. Therefore, an increase in [Pi]_In_ decreases InsP8 degradation, increases InsP8 binding to SPX, and increases Pi efflux, completing a feedback loop for [Pi]_In_ homeostasis. Published data on [Pi]_In_ by magnetic resonance spectroscopy indicate that the steady state [Pi]_In_ of skeletal muscle, heart, and brain is normally in the range of 1–5 mM, but it is not yet known whether PiT1 recycling or XPR1 activation by InsP8 contributes to Pi homeostasis in these organs. Data on [Pi]_In_ in cultured cells are variable and suggest that some cells can regulate [Pi] better than others, following a change in [Pi]_Ex_. More measurements of [Pi]_In_, influx, and efflux are needed to determine how closely, and how rapidly, mammalian [Pi]_In_ is regulated during either hyper- or hypophosphatemia.

## 1 Introduction

**FIGURE 1:**
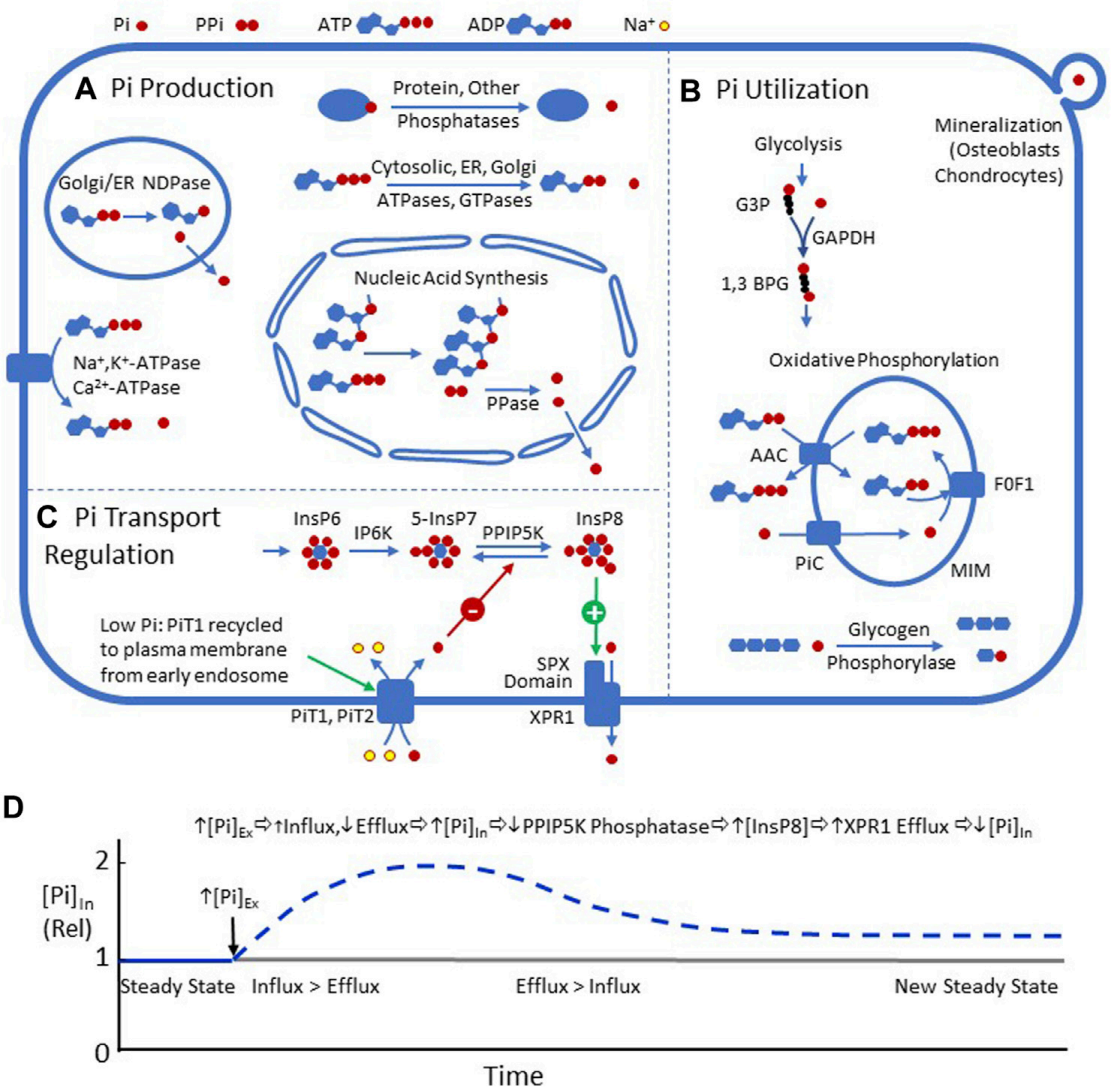
**(A)** Metabolic processes resulting in intracellular Pi production, including nucleic acid synthesis, NTPases, NDPases, and phosphatases. **(B)** Processes utilizing Pi in mammalian cells, including glycolysis (GAPDH), oxidative phosphorylation, glycogen phosphorolysis, and the formation of matrix vesicles in mineralizing tissues. **(C)** Two recently described pathways for regulating plasma membrane Pi transport. 1) PiT1 is recycled to the plasma membrane during Pi depletion (Zechner et al., 2022). 2) Efflux mediated by XPR1 is stimulated by InsP8 (Wild et al., 2016) (Gu et al., 2017) (Wilson et al., 2013) (López-Sánchez et al., 2020) (Li et al., 2020). **(D)** Hypothetical time course of [Pi]_In_, following a step increase in [Pi]_Ex_, driven by InsP8 regulation of XPR1. The increase in [Pi]_Ex_, by mass action, should decrease efflux through XPR1 and slightly increase PiT1/PiT2-mediated influx. The imbalance between influx and efflux should increase [Pi]_In_. Increased [Pi]_In_ should inhibit the phosphatase activity of PPIP5K and increase [InsP8], which binds to the SPX domain of XPR1 and stimulates efflux, resulting in a decrease in [Pi]_In_. Eventually, a new steady state should be reached with [Pi]_In_ higher than the previous state.

Inorganic orthophosphate (H_2_PO_4_^−^/HPO_4_^=^; Pi) plays numerous metabolic roles as a reactant (glycolysis, oxidative phosphorylation, glycogen phosphorolysis, and mineralization; Figure 1A) and product (nucleic acid synthesis, ATPases, GTPases, and phosphatases; Figure 1B). In addition, Pi is now recognized as a signaling molecule (Khoshniat et al., 2011; Chande and Bergwitz, 2018; Michigami et al., 2018; Abbasian et al., 2020a; Kritmetapak and Kumar, 2021). As expected for a metabolite of such central importance, plasma [Pi] is normally regulated within the relatively narrow range of 0.8–1.5 mM in adult humans by the combined actions of the kidney, intestine, bone, and parathyroid gland (Bergwitz and Jüppner, 2010; Komaba and Fukagawa, 2016; Beck and Beck-Cormier, 2020; Sun et al., 2020; Figueres et al., 2021). Less is known about intracellular Pi ([Pi]_In_) regulation in mammals.

The goal of this review is to summarize the current knowledge of the roles of plasma membrane transporters in regulating mammalian [Pi]_In_. The emphasis is on non-epithelial cells because many reviews of renal and intestinal Pi transport have been published in the past several years. This review is focused on transport and does not include several topics that are related to Pi: normal and abnormal mineralization, muscle metabolism and fatigue, polyphosphate, tumor microenvironment, nutrition, Pi toxicity, intestinal Pi transport, and non-mammalian Pi homeostasis.

## 2 Pi influx: Mammalian Na^+^-Pi cotransporters

SLC17 proteins (originally known as Type I Na^+^-Pi cotransporters) function primarily as organic anion transporters (Reimer and Edwards, 2004) and are not included in this review.

### 2.1 SLC34

Pi entry into mammalian cells is through SLC34 (Type II) and SLC20 (Type III) Na^+^-Pi cotransporters. SLC34 transporters are expressed mainly in epithelia and play a critical role in regulating plasma [Pi]. Readers are referred to several excellent reviews for discussion of the structural, functional, and pharmacological properties of SLC34 transporters (Virkki et al., 2007; Forster et al., 2013; Wagner et al., 2014; Levi et al., 2019; Hernando et al., 2021; Wagner, 2023).

### 2.2 SLC20 (PiT1 and PiT2) general properties

Type III Na^+^-Pi cotransporters PiT1 (SLC20A1) and PiT2 (SLC20A2) are widely expressed and were first identified as retrovirus receptors (Kavanaugh et al., 1994). PiT1 and PiT2 catalyze electrogenic 2 Na^+^: 1 H_2_PO_4_^−^cotransport (Virkki et al., 2007; Hernando et al., 2021). The half-maximal [Pi]_Ex_ for influx *via* PiT1 and PiT2 is ∼0.1–0.5 mM (Kavanaugh et al., 1994; Tenenhouse et al., 1998; Zoidis et al., 2004; Bøttger et al., 2006; Ravera et al., 2007; Villa-Bellosta et al., 2007). Therefore, raising [Pi]_Ex_ above 1 mM should cause only a slight increase in Pi influx through PiT1 or PiT2.

### 2.3 SLC20 pharmacology

SLC20 transporters are less sensitive than SLC34 transporters to inhibition by phosphonoformic acid (Foscarnet) (Ravera et al., 2007). Recently, the first high-affinity SLC20 inhibitor, EOS789, has been described (Tsuboi et al., 2020). Human and rat PiT1 and PiT2 expressed in CHO cells are inhibited half-maximally by 1–2 μM EOS789. SLC34 transporters are also inhibited by EOS789 but at slightly higher concentrations. The properties of EOS789 indicate that it has therapeutic potential for hyperphosphatemia associated with chronic kidney disease (Tsuboi et al., 2020; Hill Gallant et al., 2021; Doshi and Wish, 2022; Tsuboi et al., 2022). For a recent review of Na^+^-Pi cotransporter pharmacology, see Wagner, 2023.

### 2.4 PiT1 pathophysiology

Mouse PiT1 is essential for liver development, and global *Slc20A1* deletion is embryonic lethal (Festing et al., 2009; Beck et al., 2010). PiT1 knockdown decreases proliferation of HeLa and HepG2 cells, independent of PiT1 transport activity (Beck et al., 2009). High expression of PiT1 is associated with a poor prognosis in some breast cancers (Sato and Akimoto, 2017; Onaga et al., 2021). PiT1 is the main Pi transporter in vascular smooth muscle cells (Jono et al., 2000), and PiT1-mediated Pi influx is required for vascular smooth muscle cell calcification in response to elevated [Pi]_Ex_ (Li et al., 2006).

### 2.5 PiT1 regulation

Pi depletion increases PiT1 mRNA in mammalian cells by variable amounts (Kavanaugh et al., 1994; Zoidis et al., 2004; Guillén et al., 2019) but causes a large increase in plasma membrane PiT1 protein levels in HEK 293 cells (Zechner et al., 2022). With adequate Pi, PiT1 is continuously internalized and degraded; during Pi depletion, PiT1 is recycled from early endosomes to the plasma membrane by a pathway that depends on the sorting nexin SNX17 (Zechner et al., 2022). If graded recycling of PiT1 takes place under physiological conditions, it would constitute a mechanism for up-regulating Pi influx in response to hypophosphatemia.

### 2.6 PiT2 pathophysiology

Deletion of mouse *Slc20a2* results in placental calcifications and sub-viable pups (Wallingford et al., 2016), indicating a role for PiT2 in Pi transport from maternal to fetal blood. Mouse PiT2 deficiency also causes brain calcifications (Jensen et al., 2013; Ren et al., 2021), and in humans, *SLC20A2* loss-of-function mutations are associated with familial brain calcification (Wang et al., 2012; Peters et al., 2020). The effect of PiT2 deficiency on brain calcification likely results from elevated CSF [Pi], which is normally lower than plasma [Pi] because of the PiT2-dependent transport through the choroid plexus from CSF to blood (Guerreiro et al., 2014; Jensen et al., 2016).

### 2.7 PiT2 regulation

Incubation of mammalian cells in low-Pi medium causes a major increase in PiT2 mRNA and protein (Chien et al., 1997), and a low-Pi diet increases the amount of PiT2 protein in the apical membrane of the rat proximal tubule (Villa-Bellosta et al., 2009). It is not known whether PiT2 is regulated by the recycling mechanism recently described for PiT1 (Zechner et al., 2022).

### 2.8 Redundant roles of PiT1 and PiT2 in skeletal and vascular smooth muscles

In skeletal muscle, PiT2 is more highly expressed than PiT1 (Kavanaugh et al., 1994). Deletion of Pit2 alone does not affect the muscle, but knockout of both *Pit1* and *Pit2* in the mouse skeletal muscle leads to energy stress and myofiber necrosis resulting from low [Pi]_In_ (Chande et al., 2020). Therefore, Pit1 can compensate for the lack of Pit2, but either one or the other is necessary in skeletal muscle. Pit1 and Pit2 also play redundant roles in vascular smooth muscle calcification resulting from hyperphosphatemia (Crouthamel et al., 2013).

### 2.9 PiT1/PiT2 sensing of extracellular Pi

*Pit2*-knockout mice have a much smaller drop in serum FGF23 in response to a low-Pi diet than wild-type mice, indicating a role for Pit2 in sensing [Pi]_Ex_ (Bon et al., 2018a) by a mechanism involving Pit1/Pit2 heterodimerization, without the need for Pi influx (Bon et al., 2018b). Earlier work showed that Pit2 dimerization is enhanced by low [Pi]_Ex_ (Salaün et al., 2002; Salaün et al., 2004). [Pi]_Ex_ sensing by Pit1/Pit2 does not have a known role in regulating [Pi]_In_.

## 3 Pi efflux transporter XPR1 (SLC53A1)

With the normal mammalian cell Na^+^ gradient and membrane potential, the driving force for transport through PiT1 and PiT2 is inward, even if [Pi]_In_/[Pi]_Ex_ >100 (Virkki et al., 2007). [Pi]_In_ is much lower than 100 mM; therefore, in the steady state, Na^+^-coupled Pi influx is balanced by efflux. In mammalian cells other than erythrocytes, the only known Pi efflux transporter is XPR1 (SLC53A1) (Giovannini et al., 2013). XPR1 is the receptor for murine gammaretrovirus X-MLV (Battini et al., 1999), and XPR1-mediated Pi efflux is inhibited by a peptide (XRBD) derived from X-MLV (Giovannini et al., 2013).

### 3.1 Functions of XPR1

In keeping with a critical role for XPR1 in Pi efflux, mouse *Xpr1* ^−/−^ pups are not viable (Ansermet et al., 2017). Conditional knockout of mouse kidney *Xpr1* results in reduced proximal tubule Pi efflux, hypophosphatemia, and other abnormalities similar to Fanconi syndrome (Ansermet et al., 2017), which is consistent with XPR1 being the basolateral exit pathway for Pi in the proximal tubule.

Loss-of-function mutations in human *XPR1* are associated with familial brain calcification (Legati et al., 2015; Anheim et al., 2016). The fact that brain calcification is caused by LOF mutations in either *SLC20A2* (Pi influx) or *XPR1* (Pi efflux) could be explained if PiT2 is the apical and XPR1 is the basolateral Pi transporter in the choroid plexus (Wallingford et al., 2017). A defect in either transporter would reduce Pi transport from the CSF to blood and raise CSF [Pi]. However, XPR1 has not been shown definitively to be the basolateral Pi transporter in any mammalian epithelium (Wagner et al., 2019).

XPR1 is highly expressed in the placental spongiotrophoblast layer (Xu et al., 2020). In *Xpr1* ± and *Xpr1* −/− fetuses, there is reduced [Pi] in amniotic fluid and severe placental calcifications, indicating a role for Xpr1 in maternal to fetal [Pi] transport (Xu et al., 2020), as shown previously for PiT2 (Wallingford et al., 2016).

Recent work has implicated XPR1 in the tumorigenicity of ovarian cancer cells (Akasu-Nagayoshi et al., 2022; Bondeson et al., 2022), platelet polyphosphate metabolism (Mailer et al., 2021), glucose-induced Pi efflux from pancreatic β cells (Barker et al., 2021), and protection from uremic vascular calcification (Arase et al., 2022). The relationships between these processes and [Pi]_In_ homeostasis are not clear.

### 3.2 Regulation of XPR1 by inositol pyrophosphate InsP8

XPR1 has a cytoplasmic N-terminal SPX domain (Battini et al., 1999), but this is not required for Pi transport (Giovannini et al., 2013). SPX domains bind to Pi with low affinity, but they bind to inositol pyrophosphates much more tightly (Wild et al., 2016). IP6 kinases (IP6K1-2) produce 5-InsP7 from InsP6 (Wilson et al., 2013), and PPIP5-kinase (PPIP5K) produces InsP8 from 5-InsP7 (Gu et al., 2017). Knockdown of IP6K1-2 results in decreased XPR1-mediated Pi efflux and undetectable levels of 5-InsP7 and InsP8 (Wilson et al., 2019). In addition, PiT2 overexpression increases XPR1-mediated Pi efflux by a mechanism dependent on IP6K1-2 (López-Sánchez et al., 2020). These results indicate that 5-InsP7 and/or InsP8 stimulate the XPR1 transport.

PPIP5K is a dual-function enzyme with a phosphatase domain that converts InsP8 back to 5-InsP7; this phosphatase activity is strongly inhibited by Pi (Gu et al., 2017). Therefore, an increase in [Pi]_In_ inhibits the conversion of InsP8 to 5-InsP7 and increases [InsP8]. Using a combination of approaches, Shears and coworkers recently provided strong evidence that InsP8 is the dominant regulator of XPR1 (Li et al., 2020). SPX is an autoinhibitory domain in yeast low-affinity Pi transporters (Hürlimann et al., 2009; Secco et al., 2012), and it is possible that InsP8 binding stimulates transport by relieving SPX inhibition of XPR1.

These recent studies provide a new mechanistic framework for homeostasis of mammalian [Pi]_In_ (Figures 1C, D): An increase in [Pi]_In_ should lower PPIP5K phosphatase activity, increase [InsP8], and stimulate Pi efflux *via* XPR1, resulting in reduction in [Pi]_In_ toward the original level. The same pathways should also stabilize [Pi]_In_, following a decrease in [Pi]_Ex_. It is not known how many cell types share the Pi transport regulatory mechanisms, as shown in Figure 1. It is also not known how uniform [Pi]_In_ is among different mammalian cells.

## 4 Steady-state mammalian cell [Pi]_In_

### 4.1 Erythrocytes have a lower [Pi]_In_ than other mammalian cells

The erythrocyte has a Pi efflux pathway that is distinct from XPR1. Like other cells, human erythrocytes have Na^+^-coupled Pi influx (Shoemaker et al., 1988; Timmer and Gunn, 2000). Unlike other cells, red blood cell membranes have extremely high levels of the Cl^−^/HCO_3_^−^ exchanger AE1 (SLC4A1). AE1 transports Pi much more slowly than Cl^−^ (Jennings, 2021), but there is so much AE1 in red blood cells that the AE1-mediated Cl^−^-Pi exchange drives [Pi]_In_ toward the Donnan equilibrium ([Pi]_In_/[Pi]_Ex_ ∼0.6 for H_2_PO_4_^−^ and ∼0.36 for HPO_4_^=^). The balance between Na^+^-coupled influx and AE1-mediated efflux results in [Pi]_In_ lower than [Pi]_Ex_ but not as low as that expected for the Donnan equilibrium (Challa et al., 1985; Bevington et al., 1986).

### 4.2 ^31^P magnetic resonance spectroscopy

^31^P magnetic resonance spectroscopy (MRS) is a powerful method for measuring concentrations of Pi, ATP, and other metabolites *in vivo* (Meyerspeer et al., 2020). MRS is also used to measure intracellular pH (Moon and Richards, 1973; Gillies et al., 1982) and metabolic fluxes (Pesta et al., 2016). The major ^31^Pi peak in intact muscle and brain results mainly from cytosolic Pi, but a minor peak from extracellular and/or mitochondrial Pi is resolved in some studies (Kan et al., 2010). A partial list of published MRS data on [Pi]_In_ (Table 1, upper) shows that in mammalian cells other than erythrocytes, [Pi]_In_ is generally equal to or higher than [Pi]_Ex_. The MRS data indicate that [Pi]_In_ under resting conditions is ∼4 mM in skeletal muscle (highest in slow-twitch fibers) and ∼1 mM in the heart and brain.

**TABLE 1:** Partial list of published estimates of mammalian [Pi]_In_ under resting physiological conditions.

Method	Tissue	[Pi]_In_ (mM)	Source
Magnetic resonance spectroscopy	Mouse type 1, 2x	6	Kushmerick et al., (1992); Table 6
	Mouse type 2a, 2b	0.8	Kushmerick et al., (1992); Table 6
	Human soleus	6	Vandenborne et al., (1995); Table 1
	Human gastrocnemius	4.5	Vandenborne et al., (1995); Table 1
	Human gastrocnemius	5	Pathare et al., (2005); Figure 2
	Human gastrocnemius (review)	4.2	Kemp et al., (2007); Figure 1
	Mouse hind limb	2.7	Pesta et al., (2016); Figure 4A
	Rat gastrocnemius	1.6	Kasper et al., (2019); Table 1
	Dog heart	0.95	Katz et al., (1988); page H195
	Mammalian heart (review)	0.7	Glancy and Balaban, (2021); Table 4
	Human heart	1.1	Apps et al., (2021); Figure 7
	Human heart	1.1	Valkovič et al., (2022); Figure 2AB
	Human brain	0.85	Ren, J. et al., (2015); Table 1
	Human brain	0.68	Dorst et al., (2022); Figure 5
	Human brain white matter	2	Korzowski et al., (2021); Figure 7
	Human brain gray matter	1	Korzowski et al., (2021); Figure 7
	Human low-grade glioblastoma	1	Korzowski et al., (2021); Figure 7
	Human high-grade glioblastoma	1	Korzowski et al., (2021); Figure 7
	Mouse RIF-1 tumor	4	Shungu et al., (1992); Table 1
	Dwarf rat kidney	1.2	Barac-Nieto et al., (1993); page 819
	Dog proximal tubule	1.2	Chobanian et al., (1995); Table 1
EC	HeLa glucose-starved	2.1	Xu, H. et al., (2019); page 4
Extraction and colorimetry	Human erythrocyte	0.57	Bevington et al., (1986); Table 1
	Rat quadriceps	8	Brautbar et al., (1983); Table 1
	Rat heart	7.5	Brautbar et al., (1982); Table 1
	Rat renal cortex	4.1	Morris et al., (1978); Table I
	Rat liver	4.4	Morris et al., (1978); Table II
	Rat L6 myoblasts	3.2	Kemp et al., (1992); page 13
	Rat UMR 106 osteoblastic	4	Kemp et al., (1993); Figure 2
	Human Detroit 532 fibroblast	3	Kemp et al., (1993); Figure 2
	Rabbit proximal tubule	0.42	Bajaj and Baum, (1996); Figure 3
	EAhy926 EC human endothelial cells	2.4	Abbasian et al., (2015); Figure 2A
	MIN6m9 mouse pancreatic β cells	9.6	Barker et al., (2021); page 115
	HCT116 human colon tumor	14	Gu et al., (2017); Figure 3I
	HCT116 human colon tumor	2.4	Wilson et al., (2019); Figure 4A
	HCT116 human colon tumor	20.5	Li et al., (2020); Figure 1E
	MCF 10A human breast cancer	0.28	Lacerda-Abreu et al., (2021); Figure 2A
	MCF-7 human breast cancer	1.9	Lacerda-Abreu et al., (2021); Figure 2B
	MDA-MB-231 human breast cancer	0.5	Lacerda-Abreu et al., (2021); Figure 2C
	HAP1 human CML haploid	0.05	López-Sánchez et al., (2020); Figure 2G

The term “[Pi]_In_” is used here with the understanding that [Pi]_In_ is not identical to cytosolic [Pi] because the distribution of Pi between cytosol and organelles is unknown. Much of the MRS data and the [Pi]_In_ determined in single cells by a new electrochemical (EC) method were reported as mM and are listed as reported. For the human heart, [Pi]_In_ was calculated from the reported [Pi]/[PCr] ratio assuming [PCr] = 10 mM. Values for brain [Pi]_In_ are as reported in the reference or calculated from the reported ratio [Pi]_In_/[Pi]_Ex_, with the assumption that [Pi]_Ext_ in the human brain is 0.3 mM (Ren et al., 2015). Data reported as μmol/g wet weight were converted to mM (moles/L cell water) by assuming 0.68 mL H_2_O/g wet weight (Kemp et al., 2007). Data reported as nmol Pi/mg protein were converted to mM by assuming that cultured mammalian cells have 4.25 μL cell water/mg protein, derived from 0.85 μL cell water/μL cells (Model and Schonbrun, 2013) divided by 0.2 mg protein/μL cells (Milo, 2013).

### 4.3 [Pi]_In_ determined by extraction and colorimetry

[Pi]_In_ data from cultured mammalian cells obtained by extraction and colorimetry are considerably more variable (Table 1, lower), but many of the values are in the 1–5 mM range. Hydrolysis of phosphoesters during extraction, even in carefully freeze-clamped samples, likely causes overestimates of [Pi] in muscle (Meyer et al., 1985). The labile phosphoester concentrations are lower in other tissues, but ester hydrolysis is still a potential source of overestimation of [Pi] in extracts of tissues and cultured cells. In summary, physiological [Pi]_In_, in many but not all mammalian cells, is about 1–5 mM.

## 5 Ongoing questions

### 5.1 How does [Pi]_In_ change, following an increase in [Pi]_Ex_?

Although [Pi]_Ex_ is normally regulated, hyperphosphatemia is not uncommon and can result from kidney disease, high P intake, hypoparathyroidism, or tumor lysis syndrome (Davidson et al., 2004; Hruska et al., 2008; Howard et al., 2011). Elevated [Pi]_Ex_ has many adverse effects:

• Vascular calcification (Jono et al., 2000; Giachelli et al., 2005)
• Release of procoagulant microvesicles from endothelial cells (Abbasian et al., 2020b; Abbasian and Harper, 2021)
• Increased release of reactive oxygen species in a variety of cells (Lacerda-Abreu and Meyer-Fernandes, 2021)
• Premature aging (Ohnishi and Razzaque, 2010)
• Enhanced tumorigenesis (Camalier et al., 2010; Brown and Razzaque, 2018; Arnst and Beck, 2021
• Increased risk of cardiovascular events in people with coronary disease (Tonelli et al., 2005)
• Increased risk of death in critically ill patients (Zheng et al., 2022)

These effects of hyperphosphatemia are caused by a combination of [Pi]_Ex_ and [Pi]_In_. Despite a sizable body of information on [Pi]_In_ and Pi transporters, it is not known how much (and how rapidly) [Pi]_In_ changes after an increase in [Pi]_Ex_. Published data on the effects of increased [Pi]_Ex_ on mammalian or avian [Pi]_In_ show varying degrees of homeostasis:

• In rat skeletal muscle, a two-fold increase in plasma [Pi] causes a ∼33% increase in skeletal muscle [Pi] (Bevington et al., 1986).
• In UMR106 cells or Detroit 532 fibroblasts, raising [Pi]_Ex_ from 1 to 3 mM for 1 h has little effect on [Pi]_In_ (Kemp et al., 1993).
• In HCT116 cells, raising [Pi]_Ex_ from 1 mM to 5 mM for 1 hour has little effect on [Pi]_In_ (Gu et al., 2017).
• In chick tibial chondrocytes, raising [Pi]_Ex_ from 3 mM to 5 mM for 24 h causes a nearly 10-fold increase in [Pi]_In_ (Mansfield et al., 2001).
• In EAhy926 vascular endothelial cells, raising [Pi]_Ex_ from 1 to 2.5 mM for 48 h nearly doubles [Pi]_In_ (Abbasian et al., 2015).
• Raising [Pi]_Ex_ from 1 mM to 2 mM for 24 h causes a ∼2-fold increase in [Pi]_In_ in MCF10-A but a much smaller change in MCF-7 breast cancer cells (Lacerda-Abreu et al., 2021).

These data indicate that at least some mammalian cells do not maintain near-constant [Pi]_In_ when [Pi]_Ex_ is increased. Now that the InsP8 mechanism for up-regulating the XPR1 activity has been described, it will be important to determine how broadly this mechanism applies and how closely [Pi]_In_ is regulated during hyperphosphatemia in various cell types.

### 5.2 How does [Pi]_In_ change, following a decrease in [Pi]_Ex_?

Hypophosphatemia has several possible causes (genetics, malnutrition, sepsis, and dialysis) and can result in muscle weakness (Pesta et al., 2016; Romagnoli et al., 2022), erythrocyte abnormalities (Travis et al., 1971; Worley et al., 1998), and other symptoms (Gaasbeek and Meinders, 2005; Amanzadeh and Reilly, 2006). Erythrocyte vulnerability to low [Pi]_Ex_ is likely the result of Pi efflux *via* AE1 (see Section 4.1). In other mammalian cells, there are very limited data on the effect of a moderate decrease in [Pi]_Ex_ on [Pi]_In_. A 50% decrease in plasma [Pi] causes human and rat skeletal muscle [Pi]_In_ to decrease ∼20–35% (Brautbar et al., 1983; Bevington et al., 1986).

During hemodialysis, there is Pi efflux from skeletal muscle to blood (Lemoine et al., 2016; Chazot et al., 2021). *XPR1* expression is relatively low in skeletal muscle (https://www.ncbi.nlm.nih.gov/gene/9213), and it is not known whether skeletal muscle uses the XPR1/InsP8 pathway for [Pi]_In_ homeostasis or if XPR1 mediates muscle Pi efflux during dialysis.

### 5.3 How do changes in [Pi]_In_ driven by metabolism affect transport?

Changes in [Pi]_In_ can be driven by metabolism. One example is the drop in proximal tubule [Pi]_In_ associated with Fanconi syndrome of cystinosis, probably resulting from a deficiency in apical Na^+^-Pi cotransporters (Baum, 1998). Other metabolic changes in [Pi]_In_ have unknown effects on transporters:

• Exercise causes an increase in skeletal muscle [Pi]_In_ (Westerblad et al., 2002).
• Fructose loading causes a large drop in [Pi]_In_ in the kidney cortex and liver (Morris et al., 1978).
• In the perfused rat heart, insulin causes a >50% decrease in [Pi]_In_ (Polgreen et al., 1994).
• Glucose starvation causes a two-fold increase in pancreatic β cell or insulinoma cell [Pi]_In_ (Papas et al., 1997; Barker et al., 2021).

### 5.4 What is the driving force for XPR1-mediated [Pi] transport?

XPR1 expressed in tobacco leaves mediates Pi efflux (Wege and Poirier, 2014), but the transporter is difficult to express in *Xenopus* oocytes, and the nature of XPR1-mediated Pi transport is not known. Possibilities include the following:

• H^+^- Pi cotransport. The XPR1 homologs in yeast (Pho 87 and Pho90) (Hürlimann et al., 2009; Ghillebert et al., 2011) and *Arabidopsis* (PHO1) (Stefanovic et al., 2011; Chiou, 2020) are not known to be H^+^-Pi cotransporters, but an SPX-containing vacuolar Pi transporter in rice, OsSPX-MFS3, carries out the H^+^-Pi cotransport (Wang, C. et al., 2015). There is indirect evidence that XPR1 catalyzes the H^+^-Pi cotransport: stimulation of osteoclastic differentiation by RANK-L causes an increase in both XPR1 expression (Sharma et al., 2010) and H^+^-stimulated Pi transport (Ito et al., 2005; Ito et al., 2007).
• Pi-X or Pi-Pi exchange. Many transporters have partial reactions, resulting in exchange (Rosenberg and Wilbrandt, 1957; Schroers et al., 1997; Lytle et al., 1998; Russell, 2000; Glynn, 2002); the same could be true of XPR1. Exchange could be with another anion or with Pi itself. XPR1-mediated tracer efflux is stimulated by [Pi]_Ex_ (Giovannini et al., 2013; Li et al., 2020; López-Sánchez et al., 2020); *trans*-stimulation could represent the Pi–Pi exchange rather than regulatory processes. The Pi–Pi exchange could also explain the XPR1-dependent tracer Pi influx found under some conditions (Li et al., 2020). In addition, the 2:1 H_2_PO_4_^−^ -HPO_4_^=^ exchange has been proposed for proximal tubular basolateral Pi transport (Barac-Nieto et al., 2002), which may be mediated by XPR1 (Ansermet et al., 2017; Kritmetapak and Kumar, 2021).
• Pi uniporter. The mitochondrial H^+^-Pi cotransporter can be converted reversibly into a uniporter (Schroers et al., 1997), and an uncoupled Pi uniport *via* XPR1 has not been ruled out.

### 5.5 What is the basis for Na^+^-independent Pi transport that is not mediated by XPR1?

XPR1 is the only identified transporter with Pi efflux as the primary function, but there are many examples of Na^+^-independent Pi transport processes that are not known to be mediated by XPR1 in non-erythroid mammalian cells, including Caco2BBE (Candeal et al., 2014), vascular smooth muscle (Villa-Bellosta et al., 2007; Hortells et al., 2020), articular chondrocytes (Solomon et al., 2007), brain endothelial cells (Dallaire and Béliveau, 1992), and breast cancer cells (Lacerda-Abreu et al., 2019; Lacerda-Abreu et al., 2020). The genes and proteins associated with these processes are unknown. Other evidence for XPR1-independent Pi efflux is that, when XPR1 is inhibited with an XBRD peptide or expression is knocked down, Pi efflux is certainly reduced, but 25% or more of the Pi efflux does not seem dependent on XPR1 (Giovannini et al., 2013; Li et al., 2020; López-Sánchez et al., 2020). The XPR1-independent efflux could conceivably be *via* a diffusional “leak,” but it could also be mediated by a transporter that has yet to be identified.

## 6 Conclusion and perspective

In the past several years, there have been important advances in understanding the role of transporters in mammalian [Pi]_In_ homeostasis, but there is still much to learn. More data are needed on steady-state [Pi]_In_ and the time courses of changes in [Pi]_In_ in response to changes in conditions. The MRS data on skeletal muscle, heart, and brain [Pi]_In_ are reasonably consistent, but the colorimetry data on cultured cells are more variable, possibly because somewhat different procedures (timing, temperature, detergents, phosphatase inhibitors, *etc.*) were used for extraction.

Quantitative understanding of Pi transport regulation will require measurement of tracer influx and efflux in the same units under the same conditions. Tracer Pi efflux (in units convertible to moles per cell per minute) is difficult to measure because neither the intracellular Pi specific activity after tracer loading is generally unknown nor is the proportion of tracer that has been converted into organic phosphate during loading. Despite these complexities, it is possible to determine intracellular [Pi]-specific activity, following tracer loading (Kemp et al., 1992; Kemp et al., 1993); the specific activity, [Pi]_In_, and the initial rate of tracer loss could then be used to determine efflux. Measurement of both tracer influx and efflux, in the same units under the same conditions, would make it possible to determine whether net flux, following a change in [Pi]_Ex_, is caused by a change in influx, efflux, or both. This kind of experiment would make it possible to test hypotheses about how the newly described feedback mechanisms (Figure 1) regulate [Pi]_In_ in various cells under normal and pathophysiological conditions.

## Abbreviations

Pi, inorganic orthophosphate; [Pi]_Ex_, extracellular concentration of inorganic orthophosphate; [Pi]_In_, intracellular concentration of inorganic orthophosphate; PFA, phosphonoformic acid; PiT1, Na^+^-Pi cotransporter SLC20A1; PiT2, Na^+^-Pi cotransporter SLC20A2; XPR1, xenotropic and polytropic retrovirus receptor 1; SPX, suppressor of yeast gpa1 (Syg1), yeast phosphatase 81 (Pho81), and XPR1; X-MLV, xenotropic murine leukemia virus; XRBD, X-MLV receptor-binding domain; InsP6, inositol hexakisphosphate; 5-InsP7, 5-diphosphoinositol 1,2,3,4,6 pentakisphosphate; IP6K1-2, IP6 kinases 1 and -2; InsP8, 1,5 bisdiphosphoinositol 2,3,4,6 tetrakisphosphate; PPIP5K, 5-diphosphoinositol 1,2,3,4,6 pentakisphosphate kinase; PPase, pyrophosphataseSNX17: Sorting nexin 17; AAC, mitochondrial ATP/ADP exchanger; PiC, mitochondrial Pi transporter; GAPDH, glyceraldehyde-3-phosphate dehydrogenase; G3P, glyceraldehyde-3-phosphate; 1,3-BPG, 1,3-bisphosphoglycerage; MIM, mitochondrial inner membrane; F0F1, mitochondrial ATPase/ATP synthase; ER, endoplasmic reticulum; SLC, solute carrier family; MFS, major facilitator superfamily; MRS, magnetic resonance spectroscopy; EC, electrochemical.
